# Effect of Applied Stress on the Mechanical Properties of a Zr-Cu-Ag-Al Bulk Metallic Glass with Two Different Structure States

**DOI:** 10.3390/ma10070711

**Published:** 2017-06-27

**Authors:** Heng Chen, Taihua Zhang, Yi Ma

**Affiliations:** Institution of Micro/Nano-Mechanical Testing Technology & Application, College of Mechanical Engineering, Zhejiang University of Technology, Hangzhou 310014, China; hengchen@zjut.edu.cn (H.C.); zhangth@zjut.edu.cn (T.Z.)

**Keywords:** metallic glass, nanoindentation, applied stress, hardness, pop-in, creep

## Abstract

In order to investigate the effect of applied stress on mechanical properties in metallic glasses, nanoindentation tests were conducted on elastically bent Zr-Cu-Ag-Al metallic glasses with two different structure states. From spherical *P-h* curves, elastic modulus was found to be independent on applied stress. Hardness decreased by ~8% and ~14% with the application of 1.5% tensile strain for as-cast and 650 K annealed specimens, while it was slightly increased at the compressive side. Yield stress could be obtained from the contact pressure at first pop-in position with a conversion coefficient. The experimental result showed a symmetrical effect of applied stress on strengthening and a reduction of the contact pressure at compressive and tensile sides. It was observed that the applied stress plays a negligible effect on creep deformation in as-cast specimen. While for the annealed specimen, creep deformation was facilitated by applied tensile stress and suppressed by applied compressive stress. Strain rate sensitivities (SRS) were calculated from steady-state creep, which were constant for as-cast specimen and strongly correlated with applied stress for the annealed one. The more pronounced effect of applied stress in the 650 K annealed metallic glass could be qualitatively explained through the variation of the shear transformation zone (STZ) size.

## 1. Introduction

Metallic glass is scientifically defined as amorphous alloy which has a non-crystalline, but short-range order structure [1]. Due to its unique atomic configuration, metallic glass is one of the important parts of condensed matter physics. This new-structure material is promising for use in engineering fields because of its excellent mechanical properties, such as high strength, large elastic limit and good wear resistance [2,3,4]. However, the localized shear banding is dominating in plastic deformation of bulk metallic glass, causing catastrophic failure and the limited ductility severely hinders its practical application [5,6]. In order to overcome the above problems, numerous efforts have been focused on exploring new compositions in the search for ductile “perfect production”, without sacrificing high strength in the last two decades [7,8,9]. Importantly, plastic behaviors were widely studied to reveal the intrinsic deformation mechanism and to establish structural-properties correlation in metallic glasses [10,11,12]. In the last decade, a strong size effect on deformation behavior was validated in metallic glasses [13,14,15]. The plasticity could be improved remarkably without sacrificing high strength at the micro/nano scale, even combined with the transition of deformation modes (localized to homogeneous) [16]. The free volume mode and shear transformation zone (STZ) mode have been successfully applied to analyze the low-temperature deformation of metallic glasses [17,18]. Several effective methods, such as introducing crystalline secondary phase and increasing free volume content, have been developed to promote plasticity of metallic glasses [19,20]. Effects of surface treatments e.g., rolling and shot peening are also validated on metallic glasses [21,22]. Essentially, it is the modulation of shear banding events rather than changing deformation mode (localized to non-localized), which apparently increases plastic strain and delays fracture. In accordance with blocking effects of the secondary phase, residual strain/stress can be introduced into metallic glasses by surface treatments, hence suppressing both the nucleation and propagation of shear bands in metallic glasses.

The applied strain/stress effect on mechanical properties in metallic glasses has attracted many investigations that used pre-straining method [23,24,25,26,27]. From the viewpoint of engineering, it is necessary to assess the merits of surface pretreating in structural materials or anticipate material reliability under complex-stress situation. It is expected that the structure configuration would be disturbed by stress fluctuation and in turn cause alterations in the deformation mechanism. Both experiment and simulation results have reported that hardness [23], yield stress [24], creep flow [25] and shear banding morphology [26] were closely related to the type and magnitude of applied strain/stress. Using atomistic modeling, free volume evolution was speculated under applied strain/stress and expected to lead to the difference of mechanical properties [23,24,27]. It has been revealed that more excess free volume can be created in metallic glasses which own higher atomic packing densities (lower initial free volume fraction) under elasto-static stress [28]. As a consequence, the initial structure state would play an important role on the effect of applied strain/stress on mechanical properties. To the author’s best knowledge, there has been no report hitherto that investigated applied strain/stress effect concerns with different structure states. With this in mind, a Zr-Cu-Ag-Al bulk metallic glass which has high forming ability, high yield strength and large plasticity was prepared [29]. High temperature annealing was performed to attain structure relaxation. A home-made apparatus is used to elastically bend the specimen for introducing applied strain/stress. Relying on nanoindentation technology, mechanical properties can be studied at small regions which are subjected to various applied strain/stress. Due to its high accuracy, the variation of mechanical properties on the applied stress can be obtained correctly in instrumented nanoindentation and in turn the residual stress can be extracted [30,31]. In the present work, we aim to study the effect of applied strain/stress on mechanical properties and their correlation with structure states in metallic glasses.

## 2. Materials and Methods

Zr_46_Cu_37.6_Ag_8.4_Al_8_ alloy ingots were prepared from high pure elements (99.99%) by arc mixing in a Ti-gettered argon atmosphere. Alloy sheets with a rectangular cross-section of 2 mm × 10 mm were obtained by injecting alloy melt into the copper mold. The as-cast specimens with a dimension of 1 mm × 2 mm × 10 mm were cut for structure characterization and mechanical testing. The annealing was performed at the chamber of magnetron sputtering with an ultra-low base vacuum and argon protective atmosphere. The specimen was held at 650 K for 1 h and cooled inside the furnace to room temperature. Prior to the nanoindentation testing, the side surface (1 mm × 10 mm) of the specimen was carefully polished to a mirror finish. The structures of both as-cast and annealed Zr-Cu-Ag-Al specimens were detected by X-ray diffraction (XRD) with Cu *Kα* radiation. The differential scanning calorimetry (DSC) tests with heating rate of 20 K/min were performed to study the annealing effect on structure relaxation. By means of X-ray energy dispersive spectrometer (EDS) attached to the SEM, the chemical composition was detected, which is equal to the alloy ingot.

**Figure 1:**
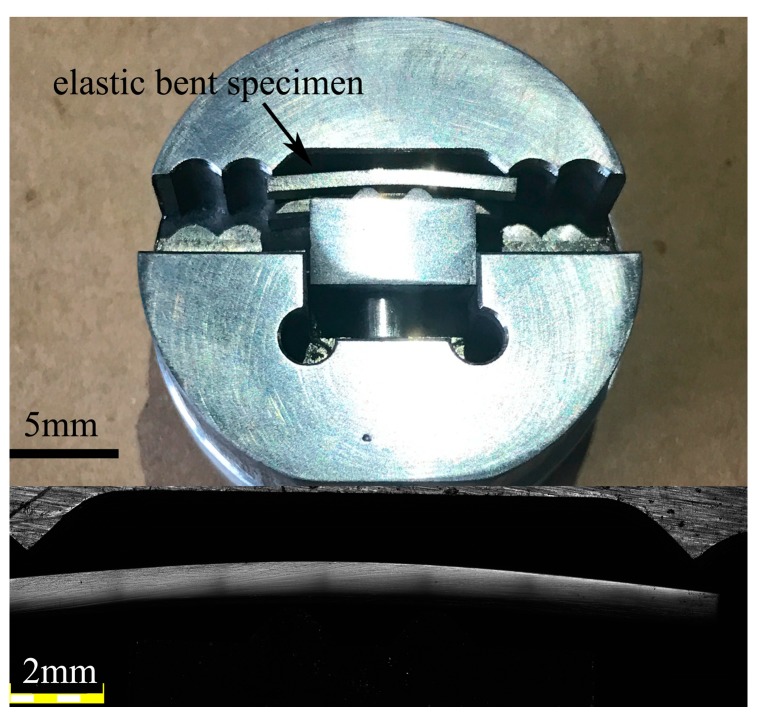
A home-made apparatus is used to elastically hold the specimen and the bending curvature is calculated by optical microscope.

Applied stress was introduced by four-point bending through a home-made apparatus, as exhibited in Figure 1. The bending curvature *r* of specimen was precisely computed as 20 mm from the optical microscope image. The applied strain could be roughly estimated as *z*/*r*, in which *z* is the distance from the selected location to the middle line of specimen. The nanoindentation tests were performed at five regions with applied strains as −1.5%, −0.75% (compressive side is referred as *z* < 0), 0% (the neutral plane), 0.75% and 1.5% (tensile side is referred as *z* > 0) for both specimens, by selecting the locations at a distance of 0.3 mm and 0.15 mm away from the neutral line, as listed in Table 1. The corresponding applied stress could be estimated as −1.39, −0.69, 0, 0.69 and 1.39 GPa, for the elastic modulus was reported as 92.4 GPa for the as-cast Zr_46_Cu_37.6_Ag_8.4_Al_8_ [29]. The yield stress of the as-cast specimen is about 1.8 GPa, which is apparently higher than the applied stress. It should also be noted that the 24 h-bent specimens can fully recover after unloading. In the following, we use “applied strain” to denote the five measured regions for simplicity.

The nanoindentation experiments were conducted at a constant temperature of 20 °C on Agilent Nano Indenter G200. Elastic modulus, hardness and information of first pop-in were studied in load-displacement (*P* vs. *h*) curves upon a special indenter, with a nominal radius of 5 μm. The maximum load was 20 mN for as-cast specimen and 25 mN for annealed one, respectively. The loading rate was constant as 0.5 mN/s. At least 25 independent measurements were conducted at each position for both specimens. The creep tests were performed by a constant load holding method upon a standard Berkovich indenter, in which the displacement of the indenter pressed into the surface at a prescribed load was continuously recorded. The duration was 500 s at a maximum load of 100 mN and the loading rate was fixed as 1 mN/s. The reliability of the creep results was confirmed by conducting 12 independent measurements. The nanoindentation tests were carried out until thermal drift reduced to below 0.03 nm/s. Furthermore, drift correction which was calibrated at 10% of the maximum load during the unloading process would be strictly performed.

## 3. Results and Discussion

### 3.1. Structure Characterization

**Figure 2:**
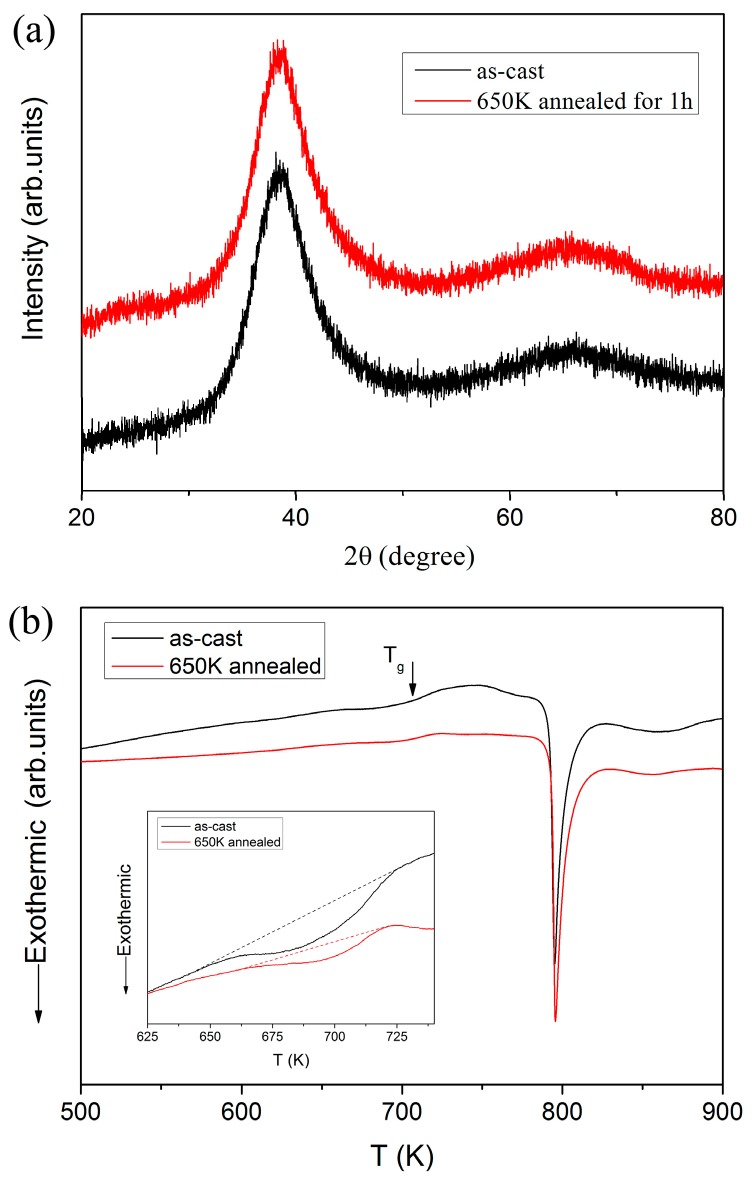
(**a**) Typical XRD patterns and (**b**) differential scanning calorimetry (DSC) curves for the as-cast and 650 K annealed Zr-Cu-Ag-Al bulk metallic glass, as well as the details of the sub-T_g_ regions of the DSC traces.

Figure 2a shows the typical X-ray diffraction patterns of as-cast and 650 K annealed Zr_46_Cu_37.6_Ag_8.4_Al_8_ specimens. It is clear that only a broad diffraction peak can be detected in each specimen, which represents a crystal-free structure. Figure 1 shows the DSC curves in which the glass transition temperature T_g_ can be observed at about 710 K, as the arrow indicates [29]. As the adopted composition has been systematically studied and confirmed to have strong glass forming ability, the XRD pattern and DSC curve may be enough to confirm the amorphous nature for both as-cast and annealed specimens without further detection by transmission electron microscopy (TEM). The inset of Figure 2b shows an enlargement of the sub-T_g_ region for the DSC curves, which corresponds to the enthalpy released during structure relaxation and can be strongly linked with the initial free volume content. It clearly shows that the structure relaxation process was more pronounced in the as-cast specimen compared to the 650 K annealed one, confirming the effect of annealing on eliminating free volume.

### 3.2. Elastic Modulus and Hardness

**Figure 3:**
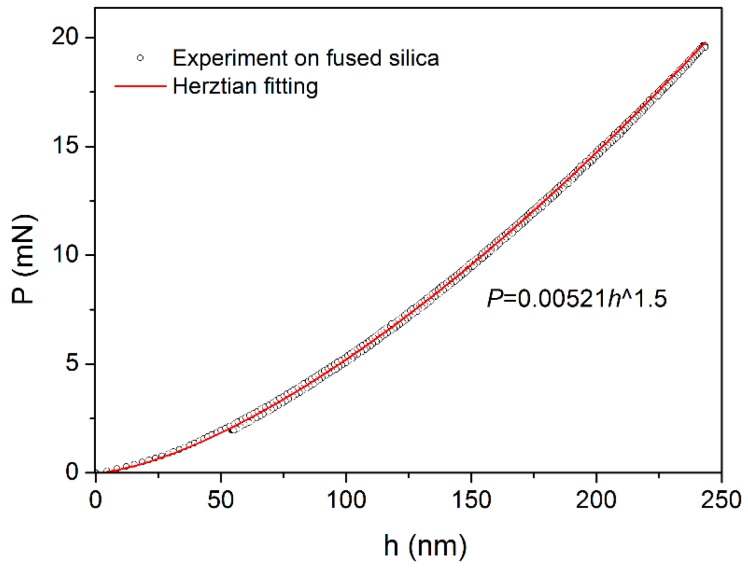
Elastic *P-h* curve in the standard fused silica upon a spherical indenter. The loading sequence can be perfectly fitted by Herztian elastic theory.

Spherical *P-h* curve in standard fused silica with loading rate of 0.5 mN/s was shown in Figure 3; the loading and unloading curves were fully overlapped, indicating an elastic deformation process. According to the Herztian elastic contact theory [32], the loading sequence could be perfectly fitted by:

(1) $$P=\frac{4}{3}E_{r}\sqrt{R}h^{1.5},$$

where *E_r_* is the reduced elastic modulus which accounts for that the elastic displacement occurs in both the tip and sample.

(2) $$E_{r}={\left(\frac{1-v_{s}^{2}}{E_{s}}-\frac{1-v_{i}^{2}}{E_{i}}\right)}^{-1},$$

*E* and *v* are the elastic modulus and Poisson’s ratio, with the subscripts s and i represent the sample and the indenter, respectively. For commonly used diamond tip, *E*_i_ = 1141 GPa and *v*_i_ = 0.07 [33], these values combined with the *E*_s_ = 72 GPa and *v*_s_ = 0.18 in fused silica can be substituted in Equation (2) to calculated the *E*_r_. Finally the effective tip radius at the front end of spherical indenter was calculated to be 2.95 μm.

**Figure 4:**
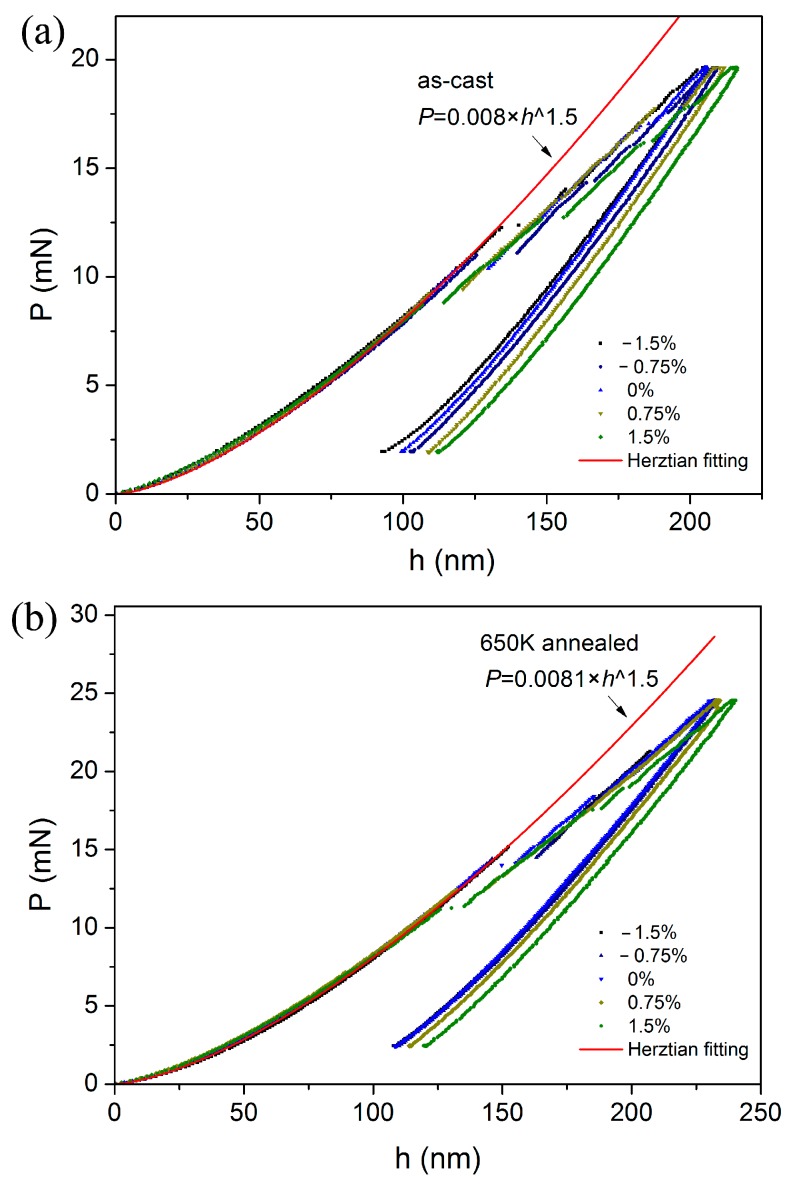
Representative spherical *P-h* curves at various pre-strained regions for (**a**) as-cast specimen and (**b**) 650 K annealed one. Pop-ins were clearly observed in each curve. Representative spherical *P-h* curves at various pre-strained regions for (**a**) as-cast specimen and (**b**) 650 K annealed one. Pop-ins were clearly observed in each curve.

Figure 4 shows the typical spherical *P-h* curves for as-cast and annealed specimens, measured at various pre-strained regions. Clearly, the *P-h* curves at the initial loading stage, namely elastic region or elastic-plastic region, were almost overlapped and can be completely fitted by the Hertzian elastic contact theory. For the as-cast specimen in Figure 4a, the power-law fitting expression is *P* = 0.008 *h*^1.5^. The Poisson’s ratio is 0.36, therefore the elastic constant can be calculated as 110.6 GPa. The elastic modulus by nanoindentation is a little higher than that reported by uniaxial compression [29]. By the same analysis, power-law fitting expression is *P* = 0.0081 *h*^1.5^ for 650 K annealed specimen in Figure 4b, and elastic modulus was deduced as 111.9 GPa. The elastic modulus was independent on applied stress and slightly increased by annealing. It may be reasonable to assume that elastic modulus is directly related to the atomic structure and belongs to the intrinsic properties of a material [25].

In a spherical-tip indentation process, hardness is defined as

*H* = *P*/2π*Rh*_c_, (3)

where *P* is the maximum load. The contact displacement *h*_c_ could be deduced as

**Figure 5:**
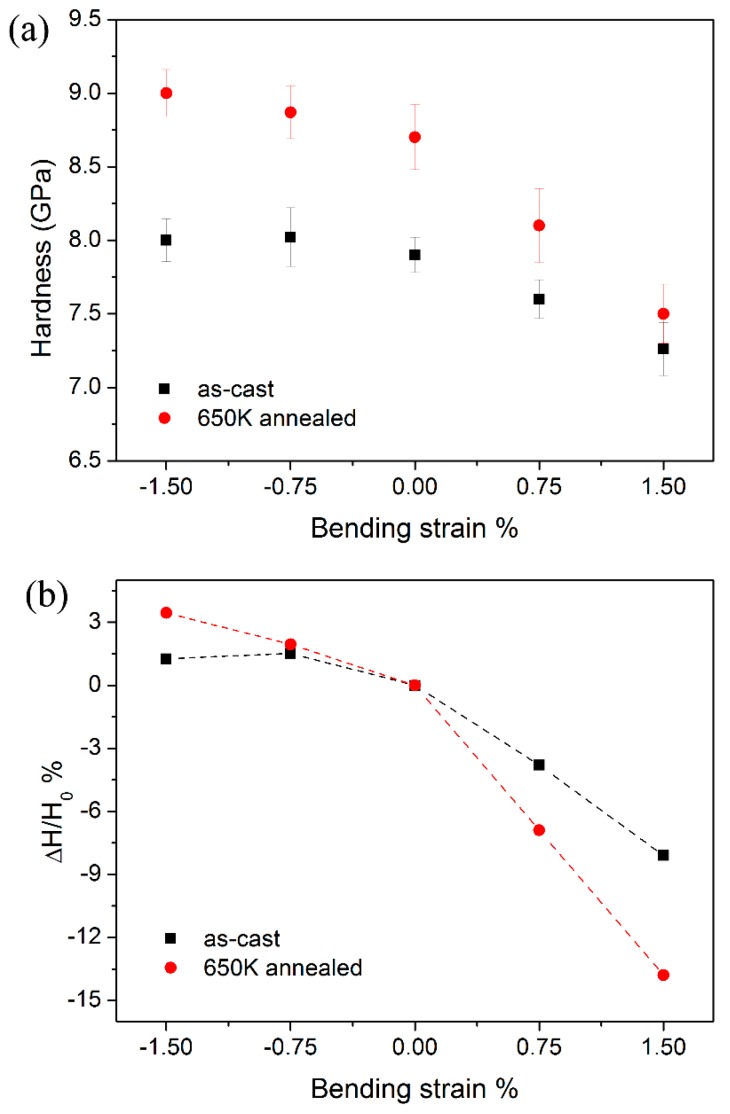
(**a**) Nanoindentation hardness *H* and (**b**) the variation trends compared to *H*_0_ in the neutral plane as a function of applied bending strans for as-cast and 650 K annealed specimens.

*h_c_* = *h* − *ε* × *P*/*S*, (4)

where *h* is the recorded indenter displacement, *ε* = 0.75 for a spherical tip, *S* is the stiffness which could be obtained from the unloading curve. The average hardness was listed in Table 1 for both specimens. At the compressive side, hardness was insensitive to the applied stress in the as-cast specimen and it slightly increased as increasing applied stress was applied in the annealed one. While at the tensile side, hardness showed a strong correlation with applied stress that dropped from 7.9 to 7.26 GPa in as-cast specimen and 8.7 to 7.6 GPa in annealed one, as the applied strain increased from 0 to 1.5%. Figure 5a clearly shows the variation trend of hardness as a function of applied strain. Moreover, hardness H obtained at the pre-strained region was compared to *H*_0_ at the neutral plane by (*H* − *H*_0_)/*H*_0_, as shown in Figure 5b. With applying 1.5% tensile applied strain, hardness reduced by ~8% and ~14% in the as-cast and annealed specimens, respectively. While at the compressive counterpart, hardness increased no more than 1.5% and 3.5% for as-cast and annealed specimens. It should be noted that the asymmetric effect of applied stress on hardness has been reported previously in metallic glasses and is well explained upon the approach of excess free volume [23]. In the present study, hardness was detected at relative shallow depth and the stress field beneath the indenter was elastoplastic, i.e., severe plastic deformation did not occur. Therefore, the influence of pile-up on the true hardness may be insignificant and the applied stress-dependent height of pile-up would not be the key factor on the herein results [26]. In addition, it is the first report that an annealing treatment may enhance the effect of applied stress on hardness at both the tensile and compressive sides.

### 3.3. Pop-in Shear Stress

Here, the pop-in events with the scale of ~10 nm can be observed in all the loading sequences. It is noted that the Herztian fitting line deviated from the exact *P-h* curve at the position of first pop-in in all the nanoindentations in Figure 4. This fact indicated the transition from elastic to elastic-plastic deformation once the first pop-in emerges, which also could be regarded as the onset of yielding under indentation [34]. According to Hertzian contact theory, the elastic contact radius is expressed as:

(5) $$a=\sqrt{Rh},$$

the maximum contact pressure beneath the indenter is defined as:

*P*_m_ = *P*/π*Rh*, (6)

the maximum shear stress *τ* at the first pop-in could represent yield stress at the onset of plasticity in nanoindentation. For a spherical indenter, the maximum shear stress of metallic glass happens at about half the elastic contact radius according to Bei’s simulation and equal to *τ* ~ 0.445 *P*_m_ [34]. The conversion coefficient is not a fixed value; while 0.31 is commonly adopted [35]. However, no matter what yield criterion is adopted, a linear function *τ* = C*P*_m_ could be expected, C is a constant related to the yield criterion. In the present work, we directly study the contact pressure of first pop-in to reveal the effect of applied stress on yield stress of as-cast and annealed metallic glasses.

**Figure 6:**
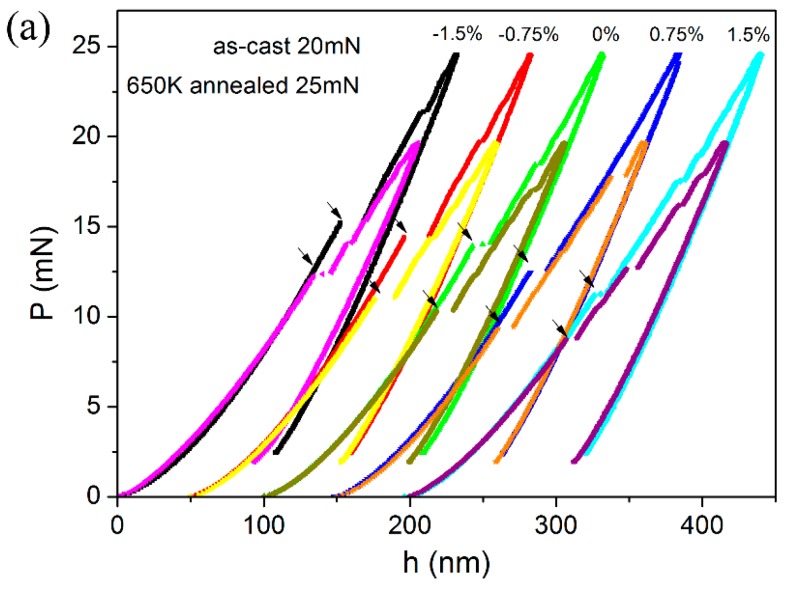

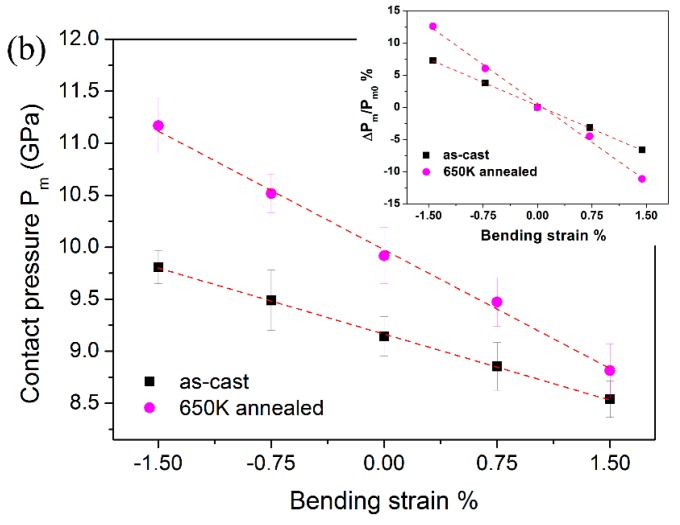
Representative spherical *P-h* curves for as-cast and 650 K annealed specimens. (**a**) Displacements of the *P-h* curves at different pre-strained regions have been offset for clearly viewing the position of first pop-in; (**b**) Contact pressure *P*_m_ were calculated from the first pop-in position and plotted as a function of bending strains.

Figure 6a shows the representative spherical *P-h* curves with various applied strains for as-cast and 650 K annealed specimens, of which the displacements have been offset for clearly viewing the positions of first pop-in. As marked by arrows, the required critical load for the first pop-in event was monotonously reduced as the decrease of the applied compressive strain and/or increase of applied tensile strain. The calculated contact pressures at first pop-in were listed in Table 1 for both specimens. In accordance with the annealing effect on hardness, the obtained contact pressure (as well as the yield stress) was effectively enhanced in the 650 K annealed specimen. As exhibited in Figure 6b, the contact pressure almost linearly decreased with an increase in the bending strain from −1.5 to 1.5% for both as-cast and annealed specimens. The strengthening effect of applied compressive stress and the softening effect of applied tensile stress on yield stress herein were found to be symmetrical. The inset in Figure 6b depicts the percentage change of *P*_m_ at the pre-strained regions compared to the neutron plane. The contact pressure was more sensitive to the applied stress in 650 K annealed specimen than the as-cast one. *P*_m_ was enhanced by ~13% by applying 1.5% compressive strain and dropped by ~12% by applying 1.5% tensile strain in annealed specimen. The percentage change of *P*_m_ in as-cast specimen was ~7% at 1.5% pre-strained regions with both stress states. Clearly, the effect of applied stress on the contact pressure (yield stress) is also structure state-dependent. Being different from the previous report [24], the obtained contact pressure was sensitive to both applied tensile and compressive stress, rather than merely at the tensile side.

### 3.4. Creep Behavior and Strain Rate Sensitivity

**Figure 7:**
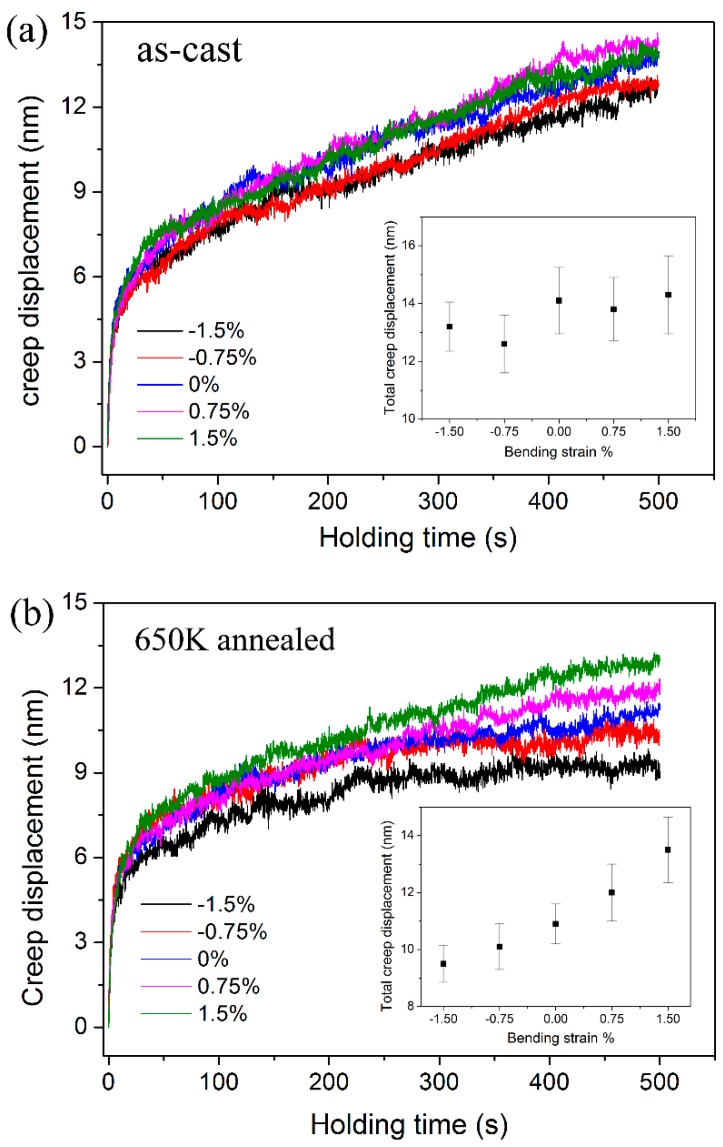
The representative creep displacements vs. holding time for (**a**) as-cast specimen and (**b**) 650 K annealed one at various pre-strained regions. The total creep displacements as a function of bending strains were exhibited in the insets.

The representative creep curves were shown in Figure 7, in which creep displacements were plotted with holding time. In order to study the creep behaviors at various pre-strained regions directly, the onset of creep deformation was set to be zero in the coordinate axis. For the as-cast specimen in Figure 7a, creep deformations recorded at various pre-strained regions exhibited little difference. And obviously, such tiny differences between the creep curves were within the range of experimental errors. For the annealed specimen in Figure 7b, creep deformation was enhanced at the tensile regions and suppressed at the compressive regions, in comparison to the creep flow at the neutron plane. The total creep displacements after 500 s holding were also summarized in the insets of Figure 5, which were plotted as a function of bending strains. The mean values of total creep displacement were in a narrow range of 12–14.5 nm for the as-cast specimen. Meanwhile, it was gradually increased from 9.5 to 13.5 nm for the annealed specimen, as the bending strain increased from −1.5 to 1.5%. In Chen’s work, it was claimed that creep displacement was roughly stable at the compressive side and increased by increasing applied tensile strain in a Zr-based metallic glass [25]. It needs to be pointed out that the total creep displacement was in a small range of 8 to 12 nm even under 400 mN holding with loading rate of 1 mN/s in Chen’s work [25]. According to their report, creep displacement was further reduced significantly under lower holding loads and/or slower loading rates. It is possible that the variation trend of creep displacement was within the range of error bars and it would be questionable to reach a “universal law” in their study. For the creep flows of annealed specimen in of Figure 7b, they completely overlapped at the transient stage and the distinction of total creep displacements resulted from the steady-state part. It is indicated that creep behaviors were intrinsically changed by applying stress in the annealed specimen.

Indentation creep has also been the most extended method to study strain rate sensitivity (SRS) in metals [36]. From indentation-creep tests, SRS can be directly obtained by applying time-displacement data. The relationship between hardness and indentation strain rate for a power-law creeping materials is

(7) $$H=A{\dot{\varepsilon }}^{m},$$

the value of SRS exponent m can be evaluated via:

(8) $$m=\frac{\partial \ln H}{\partial \ln\dot{\varepsilon }},$$

for a standard Berkovich indentation process, the strain rate $\dot{\varepsilon }$ during the holding stage can be calculated as:

(9) $$\dot{\varepsilon }=\frac{dh_{c}}{dt}\frac{1}{h_{c}},$$

and hardness is defined as:

(10) $$H=\frac{P}{24.5h_{c}^{2}},$$

the plastic displacement hc beneath a Berkovich tip could be obtained as *h_c_* = *h*-0.72 × *P*/*S*. In the current study, it is unrealistic to detect S at each recorded creep displacement. For simplicity, the *S* obtained from the creep unloading curve was adopted to calculate hardness.

**Figure 8:**
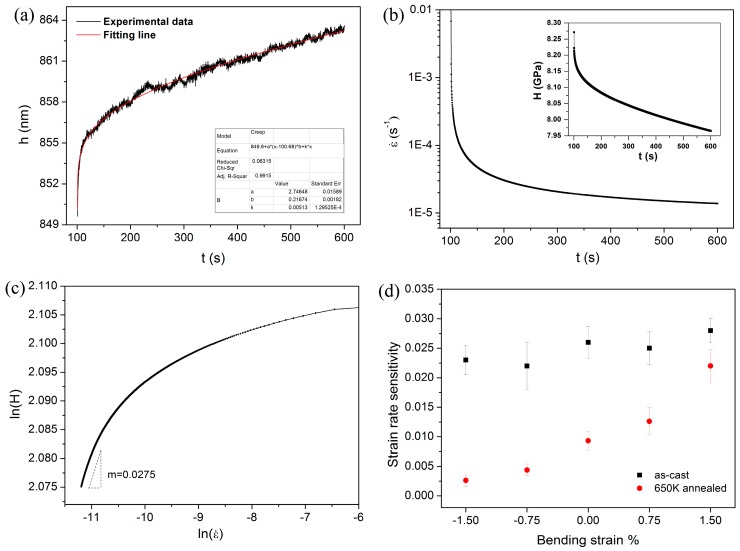
(**a**) The typical creep curve detected at the neutral plane and the fitting line for as-cast specimen; (**b**) The creep strain rate and hardness as a function of holding time; (**c**) The logar-logar correlation between hardness and strain rate for the creep deformation, strain rate sensitivities can be thus computed from the steady-state part; (**d**) The calculated strain rate sensitivities as a function of bending strain for both as-cast and annealed specimens.

The experimental data could be perfectly fitted (*R*^2^ > 0.99) by an empirical law:

*h*_(t)_ = *h*_0_ + a(*t* − *t*_0_)^b^ + *kt*, (11)

where *h*_0_, *t*_0_ are the displacement and time at the beginning of holding stage. a, b, k are the fitting constants. Figure 8a shows the typical creep curve detected at the neutral plane and the fitting line in as-cast specimen. Figure 8b shows the variation of strain rate as a function of creep time deduced from the fitting line. The creep strain rate dropped precipitously from the magnitude of 10^−2^ to 10^−4^ s^−1^ within the initial 20 s. Then it was decreased gently and fell into the range of 2 × 10^−5^ to 1×10^−5^ s^−1^ on the last 200 s duration. The variation of creep hardness is exhibited in the inset of Figure 8b. After 500 s holding, hardness reduced from about 8.3 to 7.9 GPa. Figure 8c shows the logar-logar correlation between indentation hardness and strain rate during the holding stage. SRS can be obtained as 0.0275 by linearly fitting the part of steady-state creep. For reliability, 6~8 effective creep curves were employed to reach an average value of SRS, which were listed in Table 1. They are 0.023, 0.022, 0.026, 0.025, 0.028 for as-cast specimen and 0.0026, 0.0044, 0.0093, 0.0126, 0.022 for annealed specimen, corresponding to the regions suffered −1.5%, −0.75%, 0%, 0.75%, 1.5% applied strains, respectively. Figure 8d clearly shows the correlation between strain rate sensitivity and applied bending strain for as-cast and 650 K annealed specimens. Following the rule of creep deformation, SRS was independent on the applied stress in as-cast specimen. On the other hand, the overall SRSs declined considerably after 650 K annealing. Moreover, SRS increased with increasing applied tensile strain and decreased with increasing applied compressive strain for the annealed specimen. For nanoindentation creep, the estimated value of SRS could be varied on different test conditions for a certain material. Loading rate, holding depth and indenter type are confirmed to influence the value of SRS, which could be attributed to structure change beneath the indenter [37,38,39]. Besides, the value of SRS is also relying on holding time; this smaller value would be computed at the end of a shorter duration. In the present study, we emphasized the effects of applied stress and annealing on the variation of SRS, rather than revealing the SRS characteristic or creep mechanism in metallic glasses [40]. By using the self-similar Berkovich indenter, the observed applied stress effect on creep deformation would be universal, even if it is under different holding depths and/or loading rates.

Free volume evolution as applying stress was used previously to explain the variation trends of hardness in metallic glasses that the initial free volume could be largely increased in tension and insensitive to applied compressive stress [23]. Hardness is defined as the resistance to plastic deformation, which could be closely tied to the free volume content in a metallic glass. Currently, investigation on the correlation between yield stress and applied stress is relatively scarce. To the author’s best knowledge, only Wang et al. systematically studied the effect of applied stress on the onset of yielding in metallic glass [24]. They suggested that the effective maximum shear stress at the first pop-in was essentially constant based on the distribution of shear stress beneath a spherical indenter by finite-element analysis. However, it could be subjective to merely ascribe the onset of yielding to the critical excess free volume [3]. Owing to the original work of Argon [18], the deformation unit with a local rearrangement of atoms, also referred as to shear transformation zone (STZ), has been widely applied to analyze the occurrence of plastic deformation in metallic glasses. Being different from structure defect, STZ is defined by its transience, i.e., it can only be identified from the atomic structures before and after deformation. According to Johnson and Samwer [12], there needs to be a critical fraction of activated STZ beyond which yielding will occur. The energy barrier for activating an STZ is in proportion to STZ volume, i.e., smaller STZs would be more easily agitated and readily accommodated to sustain the shear strain. In recent years, the measured STZ sizes displayed a strong correlation with strength and ductility in metallic glasses [41,42]. Moreover, the STZ size of metallic glass could be associated with Poisson’s ratio and explains the critical size of deformation mode transition at nanoscale [42,43].

Undoubtedly, STZ size plays an important role on mechanical properties and plastic deformation in metallic glasses. Following the cooperative shear model (CSM) by Johnson and Samwer [12], Pan et al. successfully developed an experimental method upon nanoindentation to calculate STZ volumes and atoms involved [42]. According to Pan’s work, the STZ volume Ω can be expressed as:

*Ω* = *kT*/*C’mH*, (12)

where *k* is the Boltzman constant, *T* is the testing temperature, $C^{\prime }=\frac{2R_{0}\zeta }{\sqrt{3}}\frac{G_{0}\gamma_{C}^{2}}{\tau_{C}}{\left(1-\frac{\tau_{CT}}{\tau_{C}}\right)}^{1/2}$ which can be computed according to Johnson and Samwer’ theory, the constants $R_{0}\approx 1/4$ and ζ≈3, the average elastic limit $\gamma_{C}\approx 0.027$, $G_{0}$ and $\tau_{C}$ are the shear modulus and the threshold shear resistance of an alloy at 0K, $\frac{\tau_{C}}{G_{0}}\approx 0.036$. The value of $\frac{\tau_{CT}}{\tau_{C}}$ at certain T can be estimated on the equation $\frac{\tau_{CT}}{\tau_{C}}=1-\frac{0.016}{0.036}{\left(\frac{T}{T_{g}}\right)}^{2/3}$, T_g_ can be obtained from the DSC curve. The STZ volume for each strained region was summarized in Table 1 for both specimens. We can reach a conclusion that STZ size is roughly stable in as-cast specimen and evidently reduced with increasing applied tensile strain and/or decreasing applied compressive strain in the annealed one. It also confirms that annealing effect could enlarge the STZ size in metallic glass [35]. As mentioned earlier, the value of SRS would be changed with different testing condition by nanoindentation creep method. Therefore, it is meaningless to discuss the specific value of STZ size in the present situation. In light of the variation trend of STZ size, the more sensitive response of mechanical properties to applied stress in 650 K annealed metallic glass could be explained qualitatively. At the regions that suffered applied tensile stress, the reduced STZ size would facilitate both instantaneous and time-dependent plastic deformations. Combined with the “softening effect” of more excess free volume, hardness and contact pressure (yield stress) consequently drop more precipitously than in the as-cast specimen. At the regions that suffered applied compressive stress, larger STZs and less excess free volume induce a faster enhancement of hardness and contact pressure (yield stress) than in the as-cast specimen. For the creep flow, the experimental result suggests that the STZ evolution might be the main creep mechanism rather than the creation and annihilation of free volume [40].

## 4. Conclusions

In summary, the effects of applied stress on mechanical properties in as-cast and 650 K annealed Zr-Cu-Ag-Al metallic glasses were systematically studied upon nanoindentation. Elastic modulus, hardness, contact pressure at the onset of yielding and creep resistance were measured at various pre-strained regions. Based on the experiment results, the following conclusions can be summarized:

(1) Hardness and the contact pressure for yielding evidently decreased with the application of tensile stress. At the compressive side, contact pressure enhancement was much more significant than hardness increase. Elastic modulus was unaffected by applied stress.
(2) Creep deformation was independent of applied stress in the as-cast specimen. However, it was facilitated by applied tensile stress and suppressed by applied compressive stress in the 650 K annealed specimen. Strain rate sensitivities of the annealed specimen were also applied stress-dependent.
(3) The effect of applied stress on mechanical properties was more pronounced in metallic glass with structure relaxation. This could be intrinsically due to the change of STZ size under applied stress.

## Figures and Tables

**Table 1:** Hardness, contact pressure at first pop-in and strain rate sensitivity, STZ volume from steady-state creep at various pre-strained regions in nanoindentation.

Z Value, mm	Applied Strain, %	Hardness, GPa		Contact Pressure, GPa		Strain Rate Sensitivity		STZ Volume, nm	
		As-Cast	Annealed	As-Cast	Annealed	As-Cast	Annealed	As-Cast	Annealed
0.3	1.5	8	9	9.8	11.2	0.023	0.0026	2.58	20.3
0.15	0.75	8.02	8.87	9.5	10.5	0.022	0.0044	2.63	12.2
0	0	7.9	8.7	9.1	9.9	0.026	0.0093	2.31	5.87
−0.15	−0.75	7.6	8.1	8.9	9.5	0.025	0.0126	2.50	4.65
−0.3	−1.5	7.26	7.5	8.5	8.8	0.028	0.022	2.33	2.88
